# Corrosion Protective Film Formation on Mg Alloy AZ31 by Exposure to Dilute Selenite Solutions

**DOI:** 10.3390/ma14020286

**Published:** 2021-01-08

**Authors:** Zhiyuan Feng, Charles C. Xu, Dadi Zhang, Rudolph Buchheit

**Affiliations:** 1 School of Chemical Engineering and Technology, Sun Yat-sen University, Zhuhai 519082, China; 2 Fontana Corrosion Center, Department of Materials Science and Engineering, The Ohio State University, Columbus, OH 43210, USA; xu.1649@osu.edu (C.C.X.); zhang.2373@osu.edu (D.Z.); 3 Department of Chemical and Materials Engineering, University of Kentucky, Lexington, KY 40502, USA; Rudolph.Buchheit@uky.edu

**Keywords:** magnesium, corrosion, EIS, selenite, selenium

## Abstract

The study of protective film formation on Mg alloys by exposure to sodium selenite solutions was conducted. Anodic polarization studies, electrochemical impedance spectroscopy studies, morphological analysis, and Energy-dispersive X-ray spectroscopy were performed on AZ31 Mg alloy after coating treatment in different concentrations of sodium selenite. The corrosion resistance was improved by around 5 times compared with control. Improved resistance to localized corrosion was observed in the coatings treated by 5 mM or 10 mM sodium selenite. The protection mechanism was ascribed to the transformation of selenite to insoluble selenium, the formation of insoluble MgSeO_3_ hydrate, and polymerization of amorphous selenium.

## 1. Introduction

Magnesium alloys have attracted attention due to its high strength and low density [1,2,3]. Then have been widely used in a number of industrial sectors such as biomedical devices, the automotive industry, aerospace components, and in the field of electronics [1,4,5,6,7]. Unfortunately, Mg is a very active metal and vulnerable to corrosion attacks when exposed to aqueous environments or humid air [1,8,9]. A number of approaches like alloying, coating, surface modification, and chemical inhibition have been addressed in the R&D community recently [1,2,10,11,12,13,14,15,16,17,18]. Among these approaches, the coating provides corrosion protection without affecting mechanical properties the way alloying approaches do.

Chromate conversion coating (CCC) has been well studied and been widely recognized as one of the most efficient coatings on magnesium [19,20,21]. The prominent chemical mechanism of CCC on Mg alloys is the reduction of Cr_2_O_7_^2−^ to Cr^3+^. The additional hydrolyzation and polymerization of Cr^3+^ will contribute to a Cr-hydroxide inorganic polymer network that results in forming a film that increases corrosion resistance [20,22,23]. However, the toxicity of chromate greatly lessens its desirability as a corrosion protective coating. With growing restrictions on chromate-bearing coating across the world, the search for chromate-free low toxic systems for magnesium alloys arises [24].

Extensive research has been conducted in order to find a high-performance coating to substitute the highly toxic chromate [25,26,27]. Common corrosion protective coatings for Mg alloys include those based on fluoride, phosphate, calcium, and rare earth metals [27,28,29,30,31]. However, the study of corrosion protection by selenite is very limited. Bengough and Whitby patented an acidic selenite-based coating bath to treat Mg alloys in the 1930s, and this is believed to be the first published report of the corrosion protection capabilities of selenite on magnesium [14,32]. Recently, an in-depth inhibition study of selenite on AZ31 was performed by Feng et al. which indicated selenite having an excellent corrosion protection performance [14]. The success inhibition study cast a possible selenite protective film study.

Compared to chromate, selenite is much less toxic. The medical study of the lethal dose of sodium selenite (LD_50_) and selenium for rats is 7 mg Se/kg body wt. The non-metal Se is believed as the reduction product of selenite having a LD_50_ of 6700 mg/kg for rats [33]. Additionally, based on the data from Occupational Safety and Health Administration (OSHA), the industry exposure limit for NaCl, selenium and its compound, and chromate are listed in Table 1 [34]. Although selenite poses some risks to human health, it appears to be practical for use in industrial applications if its exposure rate is kept at a safe level and it is much safer than chromate.

The study of protective film formation on Mg alloys by exposing to sodium selenite solutions was investigated. It was an important first step in demonstrating an approach to the future selenite-based conversion coating. The goal of this research was to study the film protection efficiency and mechanism under immersion in sodium chloride solution. The results and conclusion were important criteria to exam if it could be a good candidate chemical for the industrial coating to substitute the toxic chromate. In this study, Electrochemical measurements were used to characterize the extent of corrosion properties, scanning electron microscopy (SEM) and Energy-dispersive X-ray spectroscopy (EDS) were used to characterize the protective films.

## 2. Materials and Methods

A commercial AZ31 Mg alloy sheet (2.5–3.5 Al, 0.7–1.3 Zn, and 0.2 Mn wt.%) was used for all experiments. The alloy sheet was cut into 40 mm× 40 mm pieces and polished using silicon carbide (SiC) paper, with ethanol as a lubricant, starting from 600 and finishing with 1200 grit. The polished samples were ultrasonically cleaned in ethanol and were then dried using a compressed air gun. A selenite coating was formed by immersion in a bath containing Na_2_SeO_3_ (1 mM to 10 mM) at room temperature for 2 h. During the coating process, a stirrer bar was employed to remove the bubbles attached to the surface. All coated samples were aged in air at room temperature for 24 h before electrochemical testing.

Electrochemical experiments were all conducted in a 0.1 M NaCl solution. A traditional three-electrode vertical cell was employed for all tests. An area of 1 cm^2^ (face up) was exposed in the cell. The sample surface area to bath volume was 1 cm^2^ to 150 mL. A platinum mesh was used as the counter electrode and a saturated calomel electrode (SCE) was used as the reference electrode. Anodic potentiodynamic polarization curves were measured from −0.05 V vs. open circuit potential (OCP) with a scan rate of 0.3 mV/s. To allow for a stable OCP, a 1-h delay was used before the anodic potential scan. All experiments were replicated a minimum of three times. Electrochemical impedance spectroscopy (EIS) tests were conducted on 0.1 M NaCl after a 60 min exposure to a 0.1 M NaCl solution. EIS data were collected at a rate of seven points per decade over a frequency range of 100 kHz to 10 mHz.

Scanning electron microscopy (SEM) was used to observe the corrosion morphology of samples after 2 h of immersion in 5 mM selenite coating bath at OCP. The operation voltage ranged from 5 kV to 20 kV, and the working distance was 10.0 mm. The chemical composition of the protective film was studied by EDS obtained with an operating voltage of 30 kV.

## 3. Results

### 3.1. Electrochemical Testing

**Figure 1:**
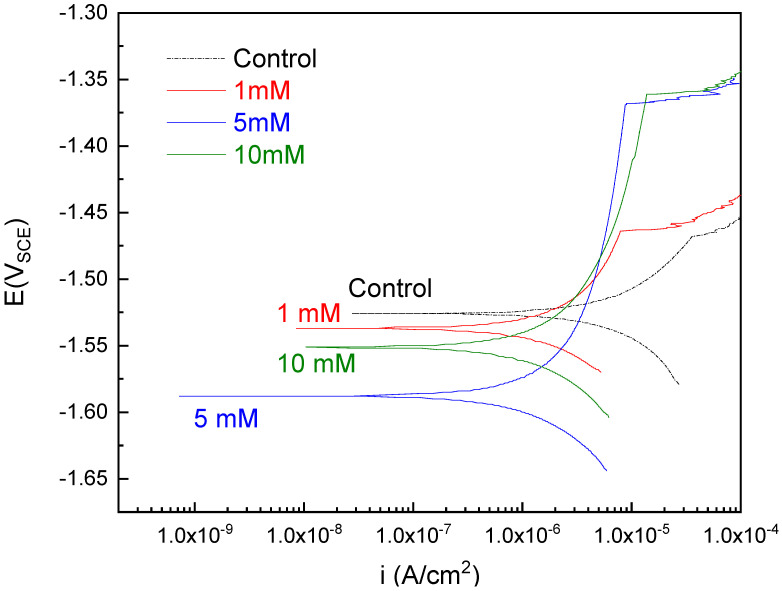
Polarization measurements were obtained in selenite coated samples or control without coating in 0.1 M NaCl.

After immersion in different coating baths with different selenite concentrations, a uniform pale grey film was formed on AZ31. In Figure 1, anodic polarization curves were collected on coated samples immersed in 0.1 M NaCl solution. The scan was collected after a 1-h OCP hold aiming to stabilize the corrosion potential. Corrosion current density values were determined by extrapolation of the linear portion of the log-based cathodic polarization curves to the intersection with corrosion potential values [14]. Breakdown potential described the breakdown of the protective film leading to a sharp increase in current density [35]. Values for the control experiment were approximately 9.0 × 10^−6^ A/cm^2^ for corrosion current density and −1.52 V_SCE_ for corrosion potential. All of the anodic polarization curves with coating exhibited a shift towards lower current density. An increase in the breakdown potential was observed in 5 mM and 10 mM samples.

**Figure 2:**
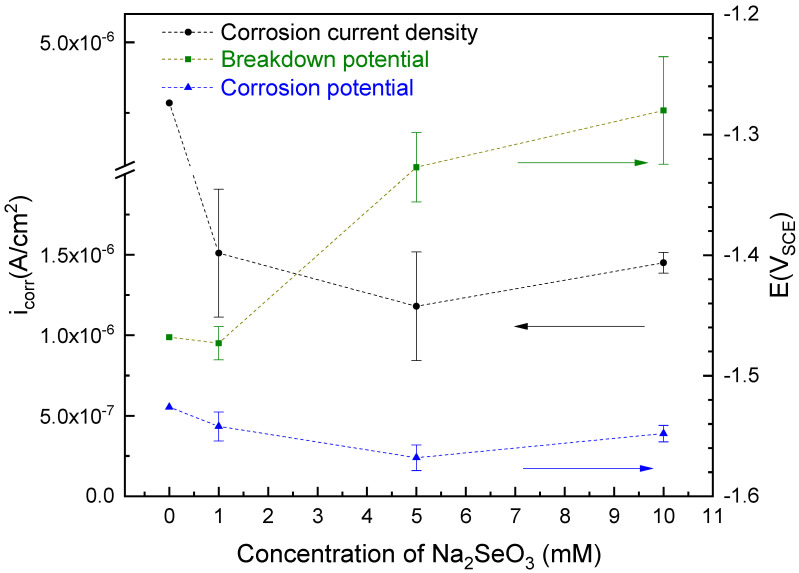
Concentration dependence of corrosion current density, breakdown potential, and corrosion potential values obtained from results of polarization tests.

After at least three repeat experiments, data were summarized in Figure 2. Suppression of anodic current depended on the concentration of selenite coating bath. As indicated in Figure 2, all samples were within the range of 1.0 × 10^−6^ to 2.0 × 10^−6^ A/cm^2^. On average, 5 mM sample had the lowest value which indicated a better corrosion resistance. This result was similar but not as good as chromate conversion coating under a similar testing environment [20]. Only a small difference was observed in the corrosion potential values for all selenite coating samples which were all at approximately −1.55 V_SCE_. The 5 mM sample was slightly lower than the others, and an upward trend in breakdown potential with increasing concentration was observed. The breakdown potential of the 1 mM sample was the lowest with results close to the control. 5 mM and 10 mM samples behaved a remarkable increase in breakdown potential.

**Figure 3:**
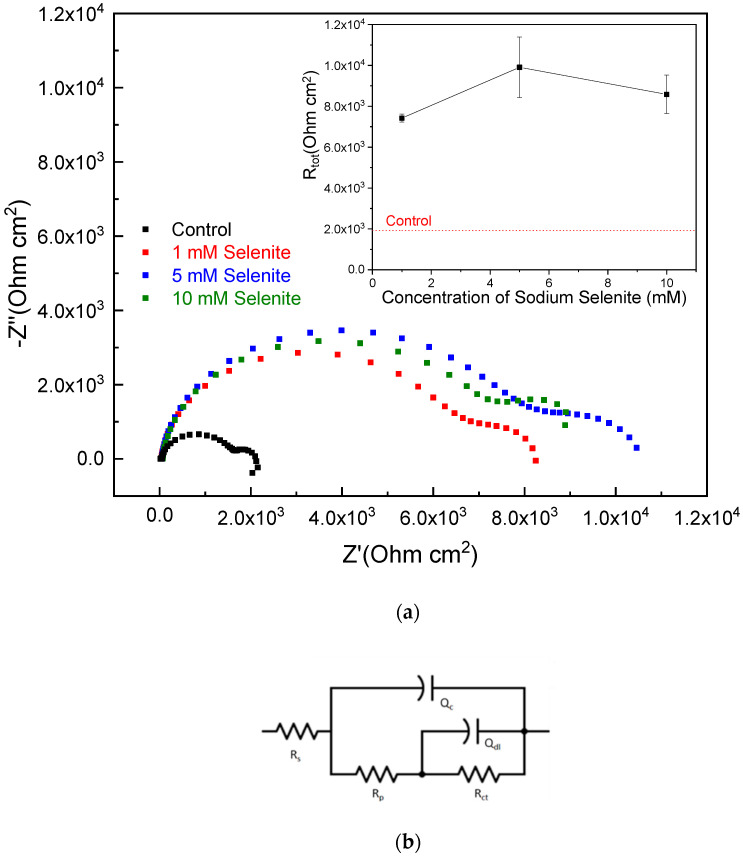
(**a**) Results of EIS testing in control without coating and various selenite coated ample in 0.1 M NaCl, (**b**) an equivalent circuit model for a defective coating.

Figure 3a illustrates the EIS Nyquist plots collected from selenium coated AZ31 samples. The EIS Bode plot can be found in supplementary materials (Figures S1–S4). AZ31 was exposed to different concentrations of selenite solutions and then measured in a 0.1 M NaCl solution after a 1-h OCP hold. An equivalent circuit model for a defective coating is shown in Figure 3b that was applied for the spectra fitting. In this model, the bulk solution resistance is represented by R_s_, R_p_ is the pore resistance representing the ohmic resistance through the coating or the corrosion product layer. Q_c_ is the reactance associated with the coating or the corrosion product layer. Q_dl_ represents the interfacial reactance that develops due to charge separation at the interface between the metal and the coating, corrosion product layer and penetrating solution, respectively [14]. The sum of R_p_ and R_ct_ represents the total resistance (R_tot_) here. All Nyquist plots in Figure 3 showed a two-time constant response. The total impedance increases significantly after applying selenium coating on AZ31 from ~2 k to ~10 k Ohm. To facilitate comparison, a summarized plot in Figure 3 shows the dependence of the total resistance measured by EIS on selenite concentrations. On average, 5 mM shows the highest R_tot_ values among others. Compared with control, it gives a 5 times increase in total resistance.

Combining the results from the potentiodynamic polarization curve and EIS, the corrosion rate data were all summarized in Table 2. It can be concluded that the data trend matches with each other. 5 mM selenite provides the best corrosion protective effect, decreasing corrosion current density, and increasing polarization resistance. Overall, these results show that while there is an increase of corrosion resistance at all selenite concentrations examined, the most protective coatings are formed at the 5 millimolar selenite concentration.

### 3.2. Surface Morphology and Characterization

**Figure 4:**
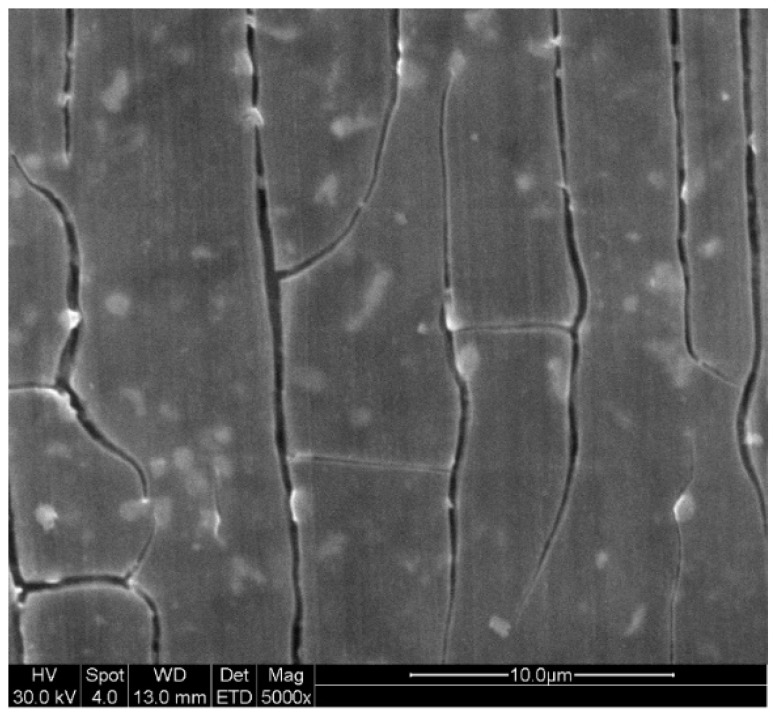
Corrosion morphology of AZ31 after 2-h immersion in 5 mM selenite coating bath.

The observed protective coating morphology was nearly identical among 1 mM, 5 mM, and 10 mM selenite immersion solution. The representative coating morphology formed by the 2 h exposure in 5 mM selenite bath, represented in Figure 4, which was similar to the previous inhibitor study and chromate conversion coating [14,20]. The shrinkage cracking phenomenon is an artifact of surface film dehydration in the microscope column. It indicates the surface films are likely a hydrated gel film that contains selenium in it which is the genesis of corrosion resistance. This gel shares characteristic of other important gel films such as chromate conversion coatings that form over a heterogeneous microstructure [19,20,21,22,23]. Its formation is most likely triggered by the reductive alkaline interfacial characteristics. The cracking on the surface was mostly in the same direction which may relate to the sample polishing defects. Several small bright sites may indicate the concentrated selenium element [14].

**Figure 5:**
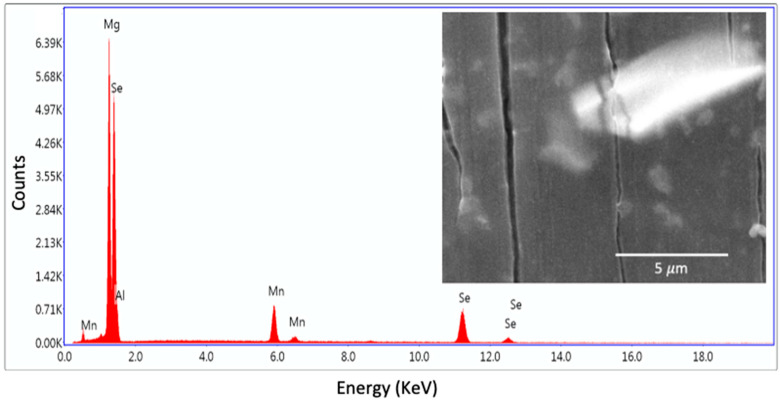
EDS elemental analysis of AZ31 after 2-h immersion in 5 mM selenite coating bath.

Energy-dispersive X-ray spectroscopy (EDS) provided an elemental analysis of protective coatings. As indicated in Figure 5, the coating consists mainly of Mg, Se, Al, and Mn. The high local intensity of Al and Mn signals come from the Al-Mn intermetallic in this study. These results indicate an intermixing of the Mg and Se through the protective coating.

## 4. Discussion

Selenite coating gave AZ31 strong corrosion protection evident by the results from the potentiodynamic polarization curve and EIS. The protection mechanism may relate to the reduction of selenite [14,36,37]. The high reactivity of magnesium results in a strong reducing agent in the aqueous environment when in contact with other reducible chemicals. In Figure 5, the EDS results indicate an intermixing of the Mg and Se through the coating. More evidence was indicated by the previous selenite inhibitor study. After immersing AZ31 in a dilute selenite bath, non-metal Se was detected by X-ray photoelectron spectroscopy (XPS) and Raman [14]. At the interface between AZ31 and coating bath, the reduction of SeO_3_^2−^ will take place. The equilibrium reaction and equilibrium reduction potential for selenite are [38]:

SeO_3_^2−^ + 6H^+^ + 4e^−^ ⇄ Se + 3H_2_O (1)

E_0_ = 0.875 − 0.0866pH + 0.0148log(SeO_3_^2−^) (2)

Based on Equation (2), the value of E_0_ associates with pH and selenite concentration. In the alkaline environment and for dissolved selenium activities ranging from 10^−6^ to 1.0, E_0_ varies from +0.27 to −0.40 V_SHE_ [38]. At the same range, the E_0_ for magnesium reduction ranges from −2.4 to −2.8 V_SHE_. Consequently, at the interface between Mg alloy substrate and selenite coating bath, there is a strong driving force for the reduction of selenite.

Another possible insoluble product arises from the following reaction [38,39,40,41,42]:

Mg^2+^ + SeO_3_^2−^ + xH_2_O → MgSeO_3_ ∙ xH_2_O (3)

Mg^2+^ will induce the precipitation of SeO_3_^2−^ to form insoluble MgSeO_3_ hydrate. This reaction was further proved from lab-synthesizing. The white bulk MgSeO_3_ hydrate was successfully made by titration of MgCl_2_ solution with Na_2_SeO_3_.

As a consequence, two insoluble products may form from the contact with Mg alloy with selenite coating bath. In the coating formation process, those products may well collaborate with the corrosion products of Mg contributing a hydrated gel protection film on AZ31.

This protective hydrated gel film is similar and can be comparable to the chromate coating [20]. For chromate coating, a reduction reaction is triggered by the reductive alkaline Mg interface. Accompanied by further hydrolyzation and polymerization process, a Cr-hydroxide inorganic polymer network is formed resulting in an excellent corrosion protection effect [20,22,23]. The reduction of selenite is able to give a similar process that relies on the unique polymerization of amorphous selenium. A selenium inorganic polymer network that possibly results in the formation of films several hundred nanometers in thickness [43,44,45]. Cooperating with other protection mechanisms, selenite has interesting and promising effects on Mg alloys.

In this study, the concentration of selenite in the coating bath had a moderate effect on the corrosion rate. The SEM morphology was nearly the same. The corrosion rate of all tests, trough 1 mM to 10 mM, were all in the same magnitude. It was revealed here that 5 mM selenite bath has the best coating performance on average which gave a decrease of corrosion current density and an increase of total resistance. Corrosion potentials were all within the range of −1.55 ± 0.03 V_SHE_. Breakdown potential was dependent on the concentration of selenite in the coating bath. Exposure in a higher concentration (5 mM and 10 mM) versus a lower concentration (1 mM) resulted in an increment of 0.14 V of breakdown potential. The breakdown potential and the corrosion potential may be assessed as the resistance to localized corrosion, with larger values indicating a lower probability of localized corrosion during free corrosion exposures [15]. Therefore, the coating in a high selenite concentration bath benefited the resistance to localized corrosion. Lower concentration did not have this property since the corrosion potential and breakdown potential were all similar to control. The thickness of the coating may affect this property. The thickness of Mg-Se mixed film relates to the bath concentration and proportional to breakdown potential which has been proven in the previous study [14].

Compared with other conventional Mg coatings in a similar testing environment, the Se-based protective film has better performance than vanadate, stannate and Ce^3+^, has similar performance with PO_4_^3−^ and Ca^2+^, and can be comparable to chromate [12,15,19,26,27,28,29,30,31,46,47]. Since the study of the selenite-based protective film was blank before. This research casts a possible selenite conversion coating study.

## 5. Conclusions

A variety of analytical methods were employed to study the performance and protection mechanism of selenite coating on Mg alloy AZ31.

1. The selenite protective film form on AZ31 upon exposure to selenite-bearing bath provides an approximate 5 times increment of corrosion resistance compared with control. A robust and apparently protective hydrated gel film was observed in SEM images collected after coating. The selenite coating from a higher concentration (5 mM and 10 mM) selenite decreases the likelihood of surface film breakdown.
2. The protection mechanism of selenite coating was the interface reaction between Mg and coating bath that results in the reduction of selenite, formation of MgSeO_3_ hydrate, and polymerization of amorphous selenium to generate a Mg-Se mixed protection film.

## Figures and Tables

**Table 1:** OSHA industry exposure limit [34].

Chemicals	Industry Exposure Limit (mg/m^3^)
NaCl	15
Selenium and its compound	0.2
Chromate	0.005

**Table 2:** Compare the results obtained from polarization data and EIS data.

Chemicals	Corrosion Current Density (A/cm^2^)	R_tot_ (Ω·cm^2^)
Control	4.57 × 10^−6^	1921
1 mM Selenite	1.51 × 10^−6^ ± 4.0 × 10^−7^	7420 ± 193
5 mM Selenite	1.18 × 10^−6^ ± 3.4 × 10^−7^	9907 ± 1480
10 mM Selenite	1.45 × 10^−6^ ± 0.6 × 10^−7^	8583 ± 940

## Data Availability

Data sharing not applicable.

## Supplementary Materials

The following are available online at https://www.mdpi.com/1996-1944/14/2/286/s1, Figure S1: Bode plot for control, Figure S2: Bode Plot for 1 mM Selenite, Figure S3: Bode Plot for 5 mM Selenite, Figure S4: Bode Plot for 10 mM Selenite.
