# Supplementary material for: Corrosion Protective Film Formation on Mg Alloy AZ31 by Exposure to Dilute Selenite Solutions
Source: Materials (Basel). 2021 Jan 8;14(2):286. doi: 10.3390/ma14020286 (PMC7827045; doi:10.3390/ma14020286)
Supplement: Supplementary file 1 [file materials-14-00286-s001.pdf]

materials

MDPI

EIS Bode Plots

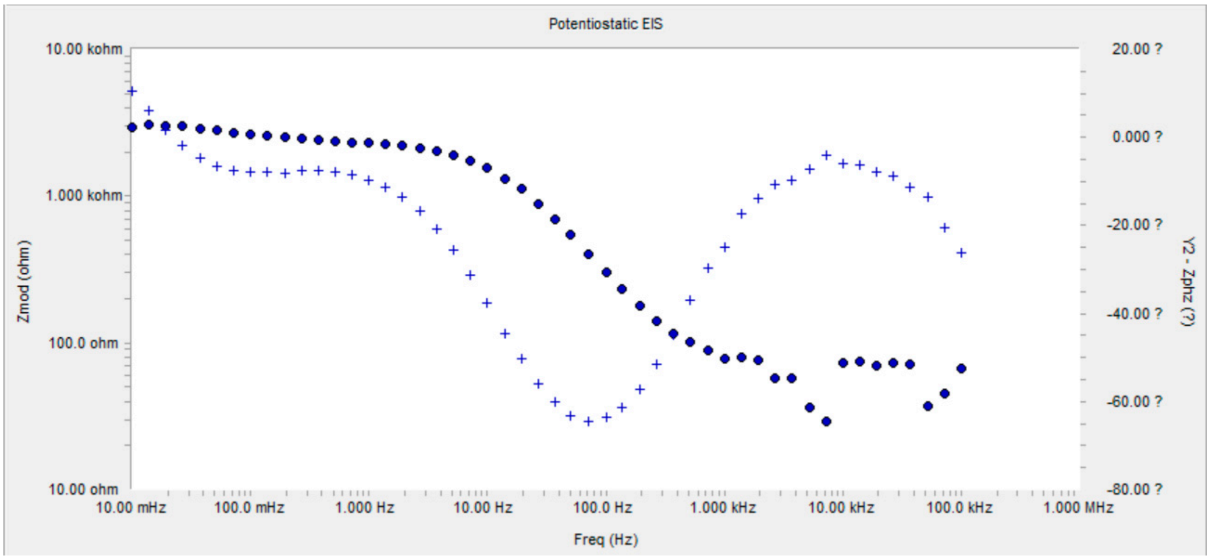

Figure S1. Bode plot for control.

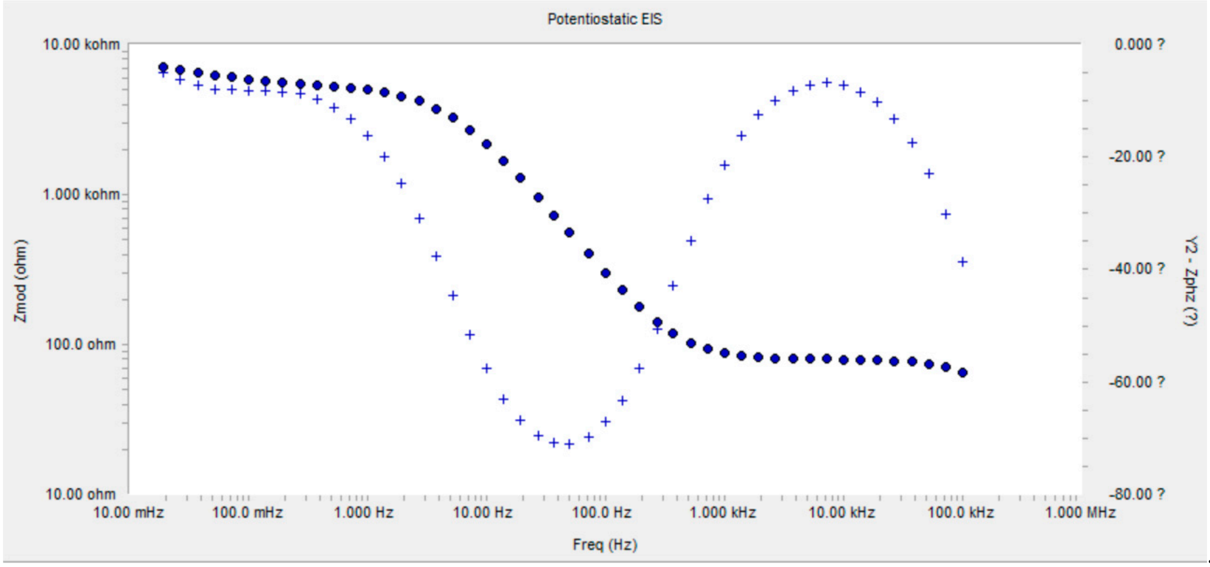

Figure S2. Bode Plot for 1 mM Selenite.

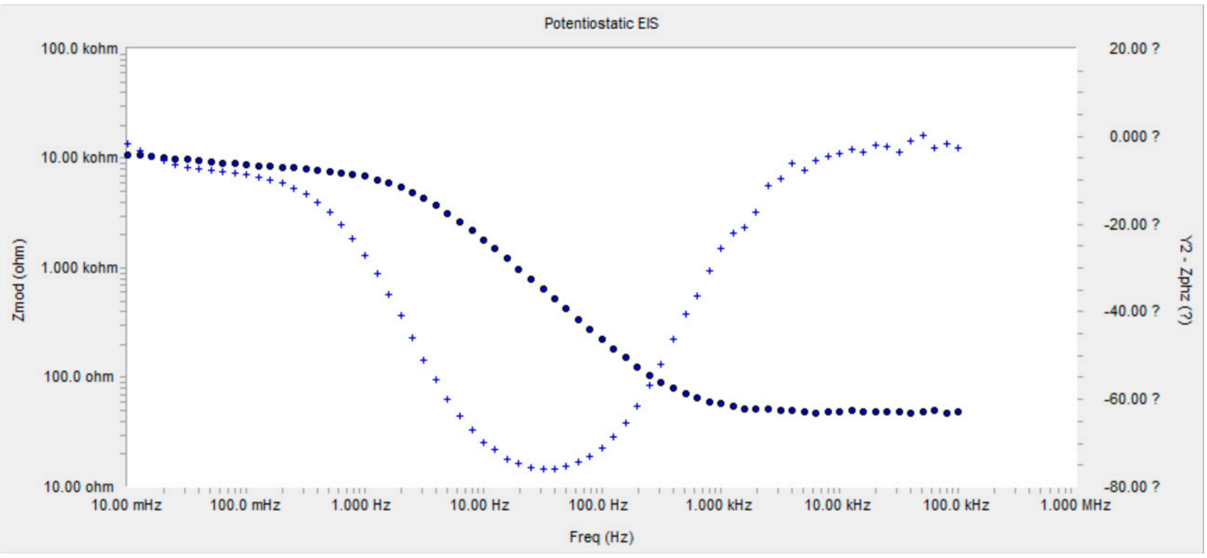

Figure S3. Bode Plot for 5 mM Selenite.

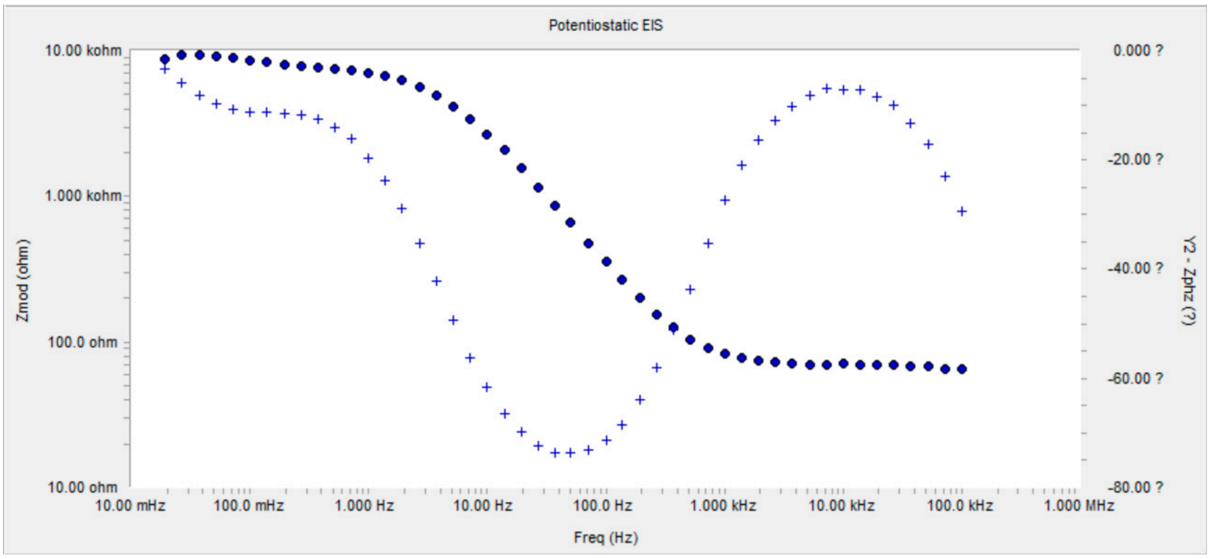

Figure S4. Bode Plot for 10 mM Selenite.
